# Endothelin-1 stimulates human monocytes *in vitro* to release TNF-α , IL-1β and IL-6

**DOI:** 10.1155/S0962935193000596

**Published:** 1993

**Authors:** E. Helset, T. Sildnes, R. Seljelid, Z.S. Konopski

**Affiliations:** 1 Department of Anesthesiology Institute of Clinical Medicine University of Tromsø Tromsø N-9038 Norway; 2 Department of Experimental Pathology Institute of Medical Biology University of Tromsø Tromsø N-9038 Norway

## Abstract

Increased plasma- and tissue levels of endothelin-1 (ET-1) during inflammatory diseases, have suggested a role of ET-1 in the pathophysiology of inflammatory reactions. The authors have studied the effect of ET-1 on cytokine release from monocytes and monocyte-derived macrophages. ET-1 increased secretion of TNF-α, IL-1β and IL-6 in a dose- and time-dependent manner. Optimal ET-1 concentration ranged from 0.01 to 1 nM. The maximal response was a 200 to 400% increase in cytokine release. A time-course study revealed that the pattern of cytokines induced by ET-1 was different in monocytes and macrophages, although an early increase in TNF-α was observed in both monocyte and macrophage supernatants. In conclusion, ET-1 stimulates monocytes and macrophages to release cytokines thereby demonstrating a potential role for ET-1 in regulation of inflammatory responses.


Mediators of Inflammation 2, 417–422 (1993)

### Endothelin-1 stimulates human monocytes *in vitro* to release TNF-α, IL-1β and IL-6

E. Helset,[1,CA] T. Sildnes,[1] R. Seljelid[2] and Z.S. Konopski[2]

[1]Department of Anesthesiology, Institute of Clinical Medicine, and [2]Department of Experimental Pathology, Institute of Medical Biology, University of Tromsø, N-9038 Tromsø, Norway

[CA] Corresponding Author

**INCREASED plasma- and tissue levels of endothelin-1 (ET-1) during inflammatory diseases, have suggested a role of ET-1 in the pathophysiology of inflammatory reactions. The authors have studied the effect of ET-1 on cytokine release from monocytes and monocyte-derived macrophages. ET-1 increased secretion of TNF-α, IL-1β and IL-6 in a dose- and time-dependent manner. Optimal ET-1 concentration ranged from 0.01 to 1 nM. The maximal response was a 200 to 400% increase in cytokine release. A time-course study revealed that the pattern of cytokines induced by ET-1 was different in monocytes and macrophages, although an early increase in TNF-α was observed in both monocyte and macrophage supernatants. In conclusion, ET-1 stimulates monocytes and macrophages to release cytokines thereby demonstrating a potential role for ET-1 in regulation of inflammatory responses.**

**Key words:** Cytokines, Endothelin-1, IL-1β, IL-6, Inflammation, Macrophages, Monocytes, TNF-α

### Introduction

Endothelin-1 (ET-1) was originally described as a potent vasoactive peptide released from endothelial cells.[1,2] The observation that macrophages and mast cells both release and have receptors for ET-1, has suggested that ET-1 possibly plays a role as a modulator of monocyte/macrophage function.[3,4]

Increased plasma- and tissue levels of ET-1 are reported in patients with inflammatory diseases such as rheumatoid arthritis, Mb.Crohn and septic shock.[5–7] Elevated plasma levels of ET-1 are even associated with the severity of illness in patients with septicaemia.[7] Furthermore, in a recent study the authors have shown that ET-1 increases microvascular permeability in isolated rat lungs provided leukocytes are present in the perfusate.[8] Against this background we hypothesized that ET-1 might play a role in the pathophysiology of inflammatory reactions. Therefore an investigation into whether ET-1 stimulation of monocytes and macrophages induced release of inflammatory mediators such as TNF-α, IL-1β and IL-6, was required. These cytokines play an important role in the development of inflammatory diseases.[9,10] Synergistic actions of these cytokines are further suggested to be of crucial importance for the outcome of septic shock.[9–11]

In the present study human monocytes and monocyte-derived macrophages were stimulated *in vitro* with ET-1 for specific time intervals, and the secretion of TNF-α, IL-1β and IL-6 in the supernatants was measured by bioassay.

### Materials and Methods

*Reagents:* Endothelin-1 (human) and endothelin antiserum were obtained from Nova Biochem (Läufelingen, Switzerland). Human rTNF-α with specific activity 2.0 × $10^7$ U/mg, monoclonal anti-human TNF-α antibodies, human rIL-6 with specific activity 2.0 × $10^8$ U/mg and monoclonal anti human IL-6 antibodies were obtained from Boehringer-Mannheim (Mannheim, Germany). Human rIL-1β with specific activity 2.0 × $10^8$ U/mg, and goat polyclonal anti-human IL-1β antibodies were purchased from British Biotechnology (Oxon, UK).

*Isolation and cultivation of monocytes and monocyte-derived macrophages:* Highly purified monocytes were obtained using the method described previously.[12] Briefly, unseparated mononuclear cells (PBMC) were isolated from buffy coat (The Blood Bank, University Hospital of Tromsø, Tromsø, Norway) by density centrifugation with Lymphoprep (Lymphoprep, Nycomed Pharma AS, Oslo, Norway). The cells were washed three times with HBSS (Gibco, Glasgow, UK) and resuspended in medium consisting of RPMI-1640 with 100 IU/ml penicillin, 100 μg/ml streptomycin, and supplemented with 25% human serum. Peripheral blood mononuclear cells (PBMC) were then seeded in 24-well culture plates (Falcon, Becton Dickinson Labware, NJ, USA) in concentration of 2 × $10^6$ cells/well.

After incubation at 37°C for 90 min the cell cultures were washed three times with pre-warmed RPMI-1640 to remove non-adherent cells. Mono-

© 1993 Rapid Communications of Oxford Ltd cyte cultures were either stimulated with ET-1 immediately, or further cultivated for 10 days to obtain monocyte-derived macrophages. The cells were assessed morphologically with Wright stain, showing >90% monocytes, and their viability was >90% as determined by Trypan blue exclusion.

*Stimulation of monocytes and monocyte-derived macrophages with ET-1:* The monocyte and macrophage cultures were incubated with various concentrations of ET-1 ranging from 0.005 nM to 100 nM or medium only (controls) for 1, 4, 8 and 24 h. To exclude endotoxin contamination, cell cultures were incubated with ET-1 denaturated by boiling for 30 min. Furthermore, in some experiments ET-1 was pre-incubated with ET-1 antiserum prior to addition to the cell cultures. For positive controls, cultures were incubated with LPS 0.1 μg/ml for 24 h. The supernatants were harvested at the time points indicated and stored at −20°C before being analysed for TNF-α, IL-1β and IL-6. Supernatants and ET-1 were tested for endotoxin using an Endospecy kit (Seikagaku Kogyo Co. Ltd, Tokyo, Japan). No significant amounts of endotoxin were detected.

*Detection of TNF-α*: TNF-α activity was determined by its cytotoxic effect on the fibrosarcoma cell line WEHI 164 clone 13, as described previously.[13] Briefly, target cells were seeded in 96-well microplates (Falcon, Becton Dickinson Labware, NJ, USA) with different dilutions of culture supernatants from ET-1 stimulated monocytes and macrophages and incubated for 24 h at 37°C. Serial dilutions of human rTNF-α (Boehringer-Mannheim) were included as a standard. The TNF-α specificity of the assay was verified using a monoclonal antibody against rTNF-α (Boehringer-Mannheim, Germany), which completely neutralized the detected activity (data not shown). Results are presented as pg/ml ± S.D. of triplicate determinations.

*Detection of IL-1β*: IL-1β activity was determined by a two-stage bioassay. The first stage involves the mouse thymocyte IL-4 NOB-1 cell line, which produces high amounts of IL-2 in response to human IL-1β.[14] Serial dilutions of human rIL-1β were included as standard. NOB-1 cells were seeded in 96-well microplates with different dilutions of culture supernatants and incubated for 24 h at 37°C. Then, 100 μl aliquots of the supernatants were transferred to a replicate 96-well microplate for the next stage. This stage of the assay involved the IL-2 dependent mouse T-cell line HT-2.[15] Aliquots (100 μl) of HT-2 cell suspension (1.5 × $10^8$ cells/well) were added to each well of the replicate microplate and incubated for a further 24 h. The IL-1β activity was neutralized by a polyclonal antibody against rIL-1β (British Bio-technology) (data not shown). Results are presented as pg/ml ± S.D. of triplicate measurements.

*Detection of IL-6:* IL-6 activity was determined using the IL-6 dependent mouse hybridoma cell line B.13.29 clone 9.[16] Briefly, serial dilutions of culture supernatants and rIL-6 as a standard, were incubated in 96-well microplates with cells (5 × $10^3$ cells/well), in a total volume of 200 μl, at 37°C for 72 h. IL-6 bioactivity was neutralized by a monoclonal antibody against rIL-6 (Boehringer-Mannheim, Germany) (data not shown). Results are presented as pg/ml ± S.D. of triplicate determinations.

*MTT assay:* Viability of the cells in the assays for TNF-α, IL-1β and IL-6 were tested by incubation with MTT [3-(4,5-dimethylthiazol-2-yl)-2,5-diphenyl-tetrazolium bromide] (Sigma, USA) as described previously.[15] Microplates were read at 570 nm ($OD_{570}$) on a NovaPath™ Mini Reader (Bio Rad, Richmond, CA, USA).

### Results

*ET-1 stimulates monocytes and macrophages to release TNF-α, IL-1β and IL-6:* There was a donor to donor variability in the levels of cytokines produced in unstimulated control cultures, as also reported by others.[17] However, the response pattern seen after ET-1 stimulation was generally the same in cultures of mononuclear phagocytes obtained from different donors. Figure 1 depicts the dose–response of ET-1-induced secretion of TNF-α (A), IL-1β (B) and IL-6 (C) from monocytes and macrophages. The concentrations of TNF-α, IL-1β and IL-6 measured in the supernatants showed an inverse U-shaped dose–response curve. The maximal cytokine production was observed in a concentration range of 0.01–1 nM ET-1. In this range the optimal ET-1 concentration appeared to be donor dependent. Furthermore, as shown in Fig. 1, monocytes secreted generally more TNF-α and IL-1β than macrophages in response to ET-1 stimulation. Denaturation by boiling or pre-incubation with ET-1 anti-serum, neutralized the effect of ET-1 on cytokine release from monocytes and macrophages (data not shown). For comparison, Table 1 shows the response to LPS 0.1 μg/ml. After 24 h, LPS stimulation caused a strong five- to ten-fold increase in release of TNF-α, IL-1β and IL-6 from monocytes and macrophages.

*Kinetics of ET-1 induced TNF-α release from monocytes and macrophages:* As shown in Fig. 2A, the time-course study revealed that ET-1 stimulation had increased monocyte TNF-α production after 1 h incubation.

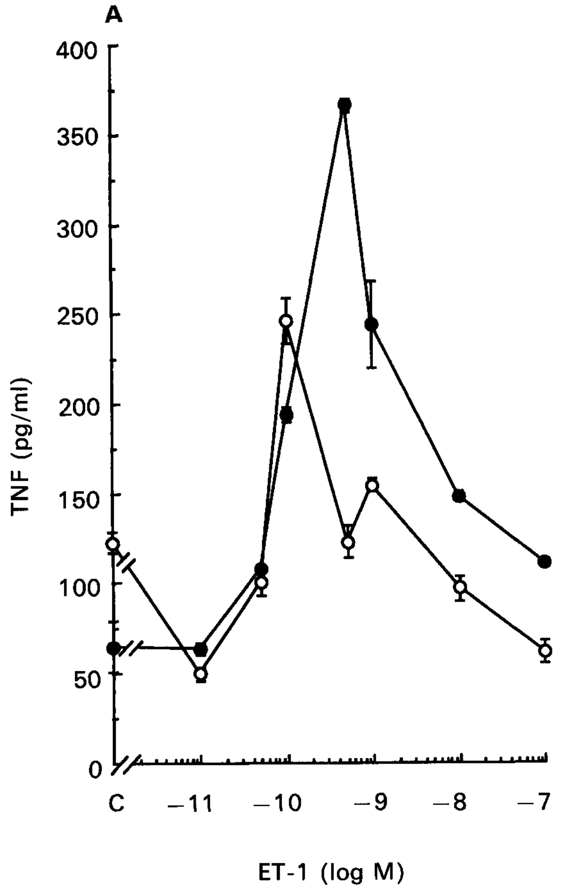

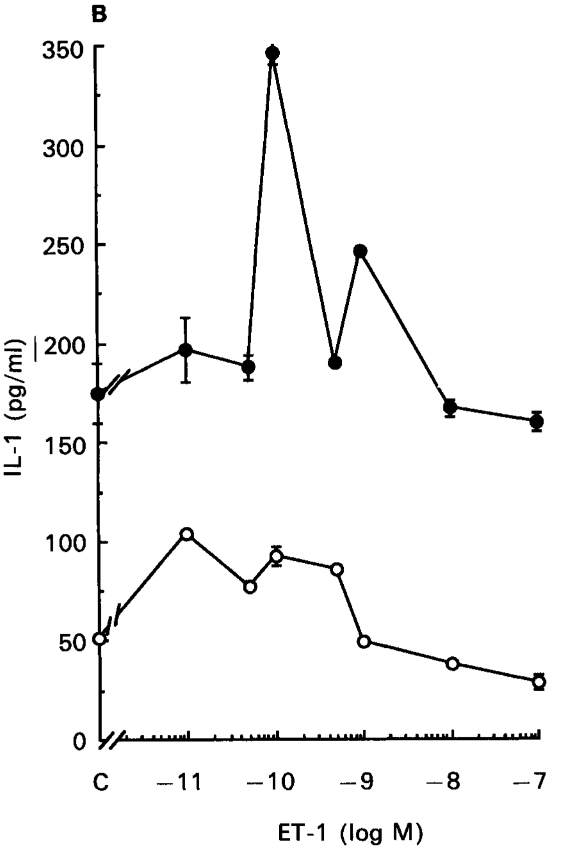

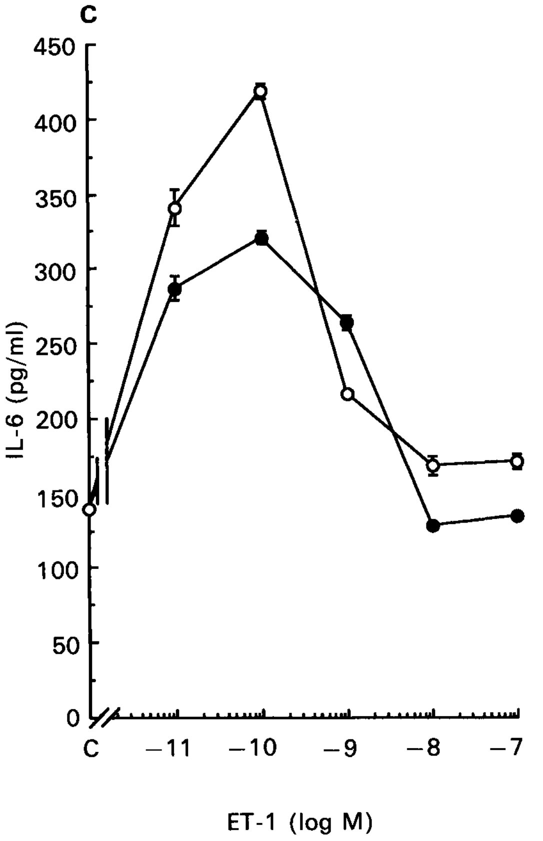

FIG. 1. Endothelin-1 (ET-1)-induced release of TNF-α (**A**), IL-1β (**B**) and IL-6 (**C**) from human monocytes and monocyte-derived macrophages. Monocyte and macrophage cultures were incubated with different concentrations of ET-1 or medium only (C: controls). Data represent the mean ± S.D. of triplicate measurements of one of three representative experiments performed in duplicate. —●—, monocytes; —○—, macrophages.

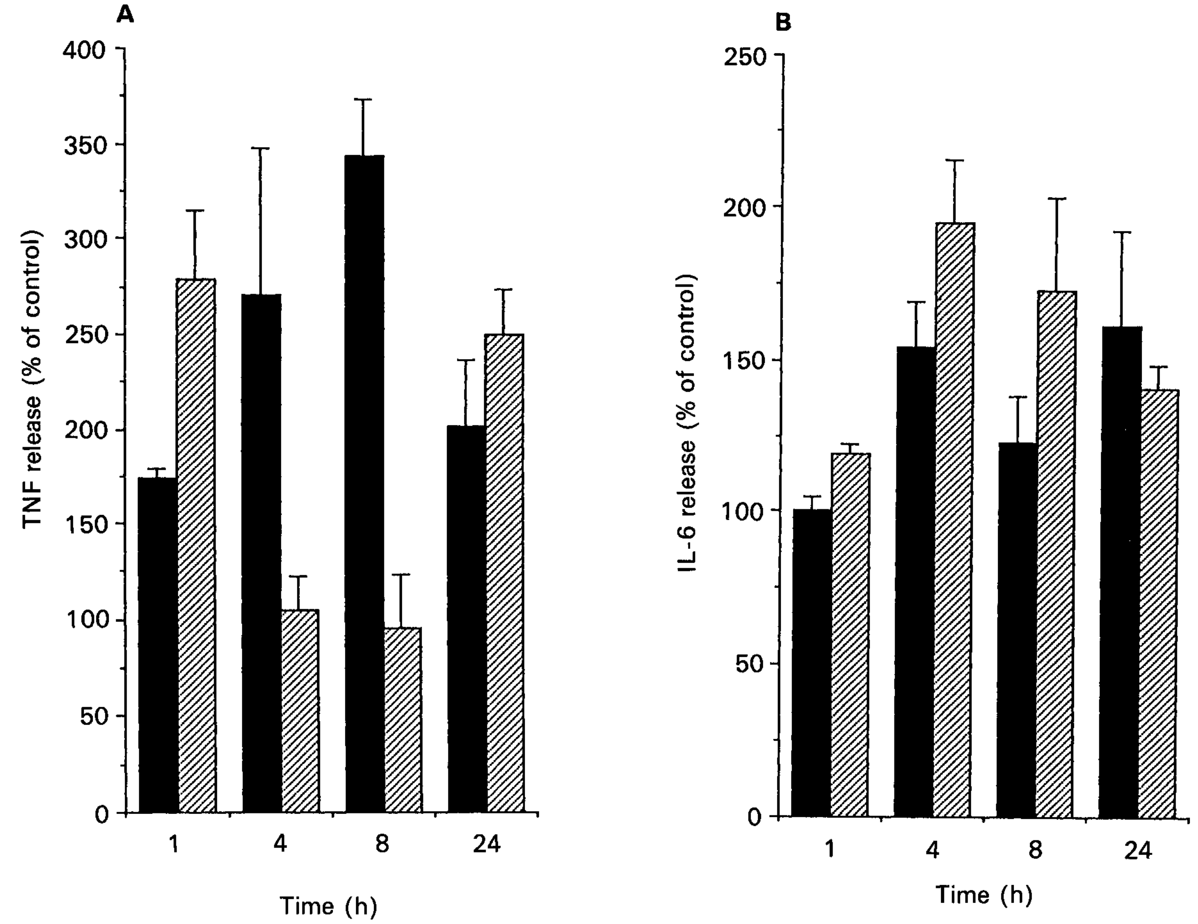

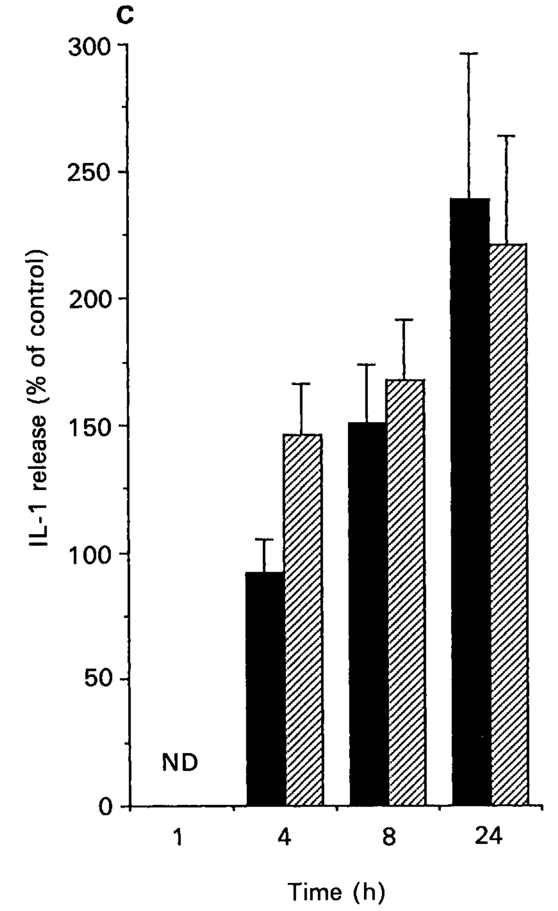

FIG. 2. The time-course study of endothelin-1 (ET-1)-induced release of TNF-$\alpha$ (**A**), IL-6 (**B**), and IL-1$\beta$ (**C**) from monocytes and macrophages in response to 0.1 nM ET-1. This ET-1 concentration was in the optimal range for maximal cytokine release as shown in Fig. 1. The monocyte and macrophage cultures were stimulated with ET-1 for 1, 4, 8 and 24 h. The results are expressed in % of controls, and given as the mean value $\pm$ S.D. of three to four experiments performed in duplicates. ■, monocytes; ▨, macrophages.

**Table 1.** LPS-induced secretion of TNF-α, IL-1β and IL-6 by monocytes and monocyte-derived macrophages[a]

| Cytokine | Cells | Controls (pg/ml) | LPS, 0.1 μg/ml for 24 h (pg/ml) |
|---|---|---|---|
| TNF-α | Monocytes | 290 ± 13 | 2008 ± 81 |
| | Macrophages | 109 ± 10 | 585 ± 70 |
| IL-1β | Monocytes | 109 ± 15 | 3876 ± 79 |
| | Macrophages | 225 ± 50 | 814 ± 13 |
| IL-6 | Monocytes | 202 ± 40 | 1226 ± 78 |
| | Macrophages | 163 ± 30 | 1246 ± 34 |

[a]The monocyte and macrophage cultures were incubated with LPS 0.1 μg/ml or medium alone (controls) for 24 h. The culture supernatants were analysed for TNF-α. IL-1β and IL-6 as described under Materials and Methods.

TNF-α production continued to increase by 4 h and reached a maximum, with three-fold increase, by 8 h.

In the macrophage culture supernatants, ET-1 had increased TNF-α production three-fold by 1 h; whereas by 4 h and 8 h the TNF-α level had returned to baseline. By 24 h the TNF-α level had increased by 250%.

*Kinetics of ET-1-induced IL-6 release from monocytes and macrophages:* As shown in Fig. 2B, ET-1 increased monocyte IL-6 production slightly after 4 h stimulation, and levels were still elevated at 24 h.

In the macrophage cultures ET-1 had induced IL-6 production by 1 h, reaching a two-fold peak concentration in the supernatants within 4 h, and declining, but still increased as compared with controls, by 24 h.

*Kinetics of ET-1-induced IL-1 release from monocytes and macrophages:* As shown in Fig. 2C, ET-1 increased monocyte IL-1β production, as measured in the supernatants, after 8 h, reaching more than a two-fold increase by 24 h. In macrophage supernatants increased IL-1β levels were detected by 4 h, reaching a maximal two-fold increase after 24 h. No release of IL-1β was detected in monocyte or macrophage supernatants after 1 h incubation time.

### Discussion

The present results show that ET-1 stimulates human monocytes and macrophages to release TNF-α, IL-1β and IL-6 in a dose- and time-dependent manner. So far, the potential of ET-1 to affect immune and inflammatory cells is largely unexplored. The only previously encountered study was done by Millull *et al.*, who demonstrated that ET-1 stimulation of alveolar macrophages increased arachidonic acid (AA) release by a maximal 300%.[18] In the present study, the same concentration range of ET-1, 0.1 to 1 nM, induced an optimal response with a 200 to 400% increase in cytokine release.

The monocyte cultures secreted generally more TNF-α and IL-1β than did the macrophage cultures, in response to ET-1. The same pattern was observed after LPS stimulation in the present study. These findings are in accordance with previous studies, concluding that the capacity of mononuclear phagocytes to secrete TNF-α and IL-1β may vary with their state of differentiation.[19,20]

Stimulation with 0.1 μg/ml LPS caused a five- to ten-fold increase in cytokine release from monocytes and macrophages. The generally lower release of cytokines in response to ET-1, indicates that LPS and ET-1 activate different mechanisms for cytokine release in mononuclear phagocytes. Denaturating ET-1 by boiling did not increase cytokine production, which excludes the possibility that the observed effect of ET-1 was caused by contamination with endotoxin.

The time-course study revealed a sequential release of TNF-α, IL-1β and IL-6 from both monocytes and macrophages. TNF-α was released prior to IL-1β and IL-6. A similar pattern was reported previously after LPS stimulation of mononuclear phagocytes *in vitro*, in septicaemia in experimental animals and in patients with meningococcal septic shock.[9,11,20] Previous reports have also documented that TNF-α, IL-1β and IL-6 might interact on the production and release of each other.[10,11,17,21] IL-6 has been reported to suppress TNF-α production in mononuclear phagocytes.[17,22] This might explain the reduction of TNF-α observed in the macrophage supernatants in parallel with the peak concentration of IL-6. In the monocyte cultures, ET-1 caused only a slight increase in IL-6 secretion, and no parallel reduction of TNF-α was observed. This also demonstrates that ET-1 induces a differential modulation of TNF-α and IL-6 secretion from monocytes and macrophages.

In experimental animals TNF-α is shown to cause a leukocyte dependent lung injury, and to increase microvascular permeability in the pulmonary circulation.[23] Thus the ET-1 induced TNF-α secretion from monocytes and macrophages as shown in the present study, might explain the earlier observation of ET-1 induced leukocyte dependent lung injury in isolated rat lungs.[8]

In patients with septic shock, plasma levels of ET-1 have been reported to be 20 pg/ml.[7] This coincides with the lower range of the ET-1 concentrations causing cytokine release from monocytes and macrophages in the present study. However, many workers favour the belief that ET-1 primarily functions in an autocrine manner, and that circulating levels of ET-1 only reflect an overflow to the circulation, indicating that local concentrations of ET-1 are substantially higher.[2]

The present observations extend the understanding of ET-1 as a vasoactive peptide, and

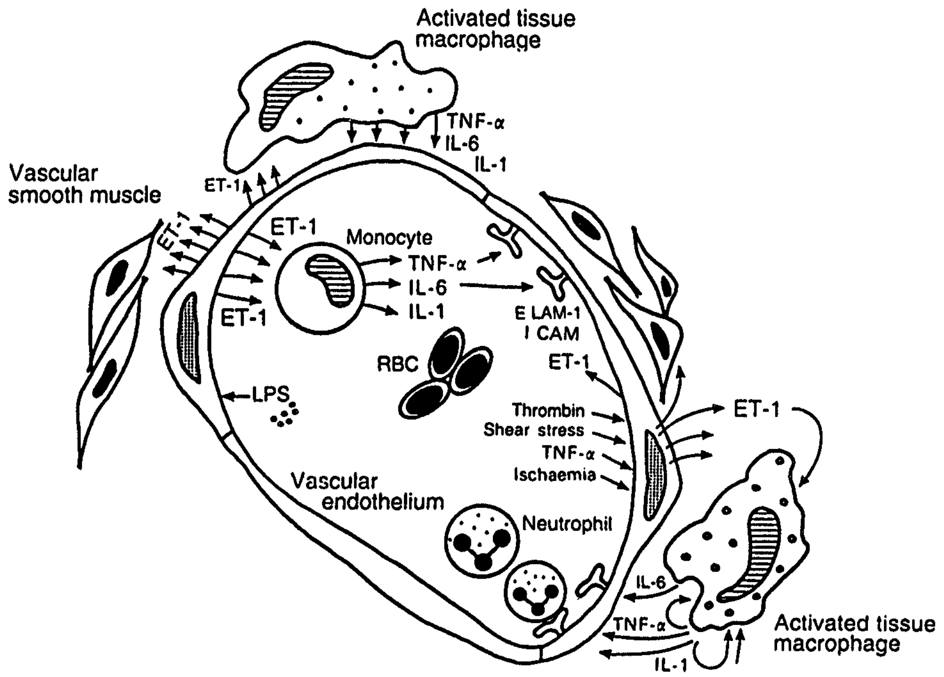

FIG. 3. Endothelin-1 (ET-1) is reported to be released from endothelial cells in response to thrombin, endotoxin, TNF-α and injury caused by ischaemia and shear stress.[2,24–27] In the present study it is documented that ET-1 stimulates monocytes and macrophages to release TNF-α, IL-1β and IL-6. These results suggest a new possibility for interactions between mononuclear phagocytes and endothelial cells.

demonstrate that ET-1 is capable of activating mononuclear phagocytes, thereby causing release of TNF-α, IL-1β and IL-6. The present results also suggest a new possibility for the interactions between endothelial cells and macrophages, as illustrated in Fig. 3.

### References

1. Yanagisawa M, Kurihara H, Kimura S *et al.* A novel potent vasoconstrictor peptide produced by vascular endothelial cells. *Nature* 1988; **332**: 411–415.
2. Lüscher TF, Boulanger CM, Dohi Y, Yang Z. Endothelium-derived contracting factors. *Hypertension* 1992; **19**: 117–130.
3. Ehrenreich H, Anderson RW, Fox CH *et al.* Endothelins, peptides with potent vasoactive properties are produced by human macrophages. *J Exp Med* 1990; **172**: 1741–1748.
4. Ehrenreich H, Burd PR, Rottem M *et al.* Endothelins belong to the assortment of mast cell-derived and mast cell-bound cytokines. *The New Biologist* 1992; **4**: 147–156.
5. Miyasaka N, Hirata Y, Ando K *et al.* Increased production of endothelin-1 in patients with inflammatory arthritides. *Arthritis and Rheumatism* 1992; **35**: 397–400.
6. Murch SH, Braegger CP, Sessa WC, MacDonald TT. High endothelin-1 immunoreactivity in Crohn's disease and ulcerative colitis. *Lancet* 1992; **339**: 381–385.
7. Pittet JF, Morel DR, Hemsen A *et al.* Elevated plasma endothelin-1 concentrations are associated with the severity of illness in patients with sepsis. *Ann Surg* 1991; **213**: 261–264.
8. Helset E, Kjæve J, Hauge A. Endothelin-1-induced increases in microvascular permeability in isolated, perfused rat lungs requires leukocytes and plasma. *Circulatory Shock* 1993; **39**: 15–20.
9. Waage A, Aasen AO. Different role of cytokine mediators in septic shock related to meningococcal disease and surgery/polytrauma. *Immunol Rev* 1992; **127**: 221–230.
10. Billau A, Vandekerchove F. Cytokines and their interactions with other inflammatory mediators in the pathogenesis of sepsis and septic shock. *Eur J Clin Invest* 1991; **21**: 559–573.
11. Fong Y, Tracey KJ, Lyle L *et al.* Antibodies to cachectin/tumor necrosis factor reduce interleukin 1β and interleukin 6 appearance during lethal bacteremia. *J Exp Med* 1989; **170**: 1627–1633.
12. Bøyum A. Isolation of mononuclear cells and granulocytes from human blood. *Scand J Clin Lab Invest* 1968; **97**: 77–89.
13. Espevik T, Nissen-Meyer J. A highly sensitive cell line, WEHI 164 clone 13, for measuring cytotoxic factor/tumor necrosis factor from human monocytes. *J Immunol Methods* 1986; **95**: 99–105.
14. Gearing AJH, Bird CR, Bristow A, Poole S, Thorpe R. A simple sensitive bioassay for interleukin-1 which is unresponsive to $10^3$ U/ml of interleukin-2. *J Immunol Methods* 1987; **99**: 7–11.
15. Mosmann TR, Cherwinski H, Bodn MW, Giedlin MA, Coffman RL. Two types of murine helper T cell clone. I. Definition according to profiles of lymphokine activities and secreted proteins. *J Immunol* 1986; **136**: 2348–2357.
16. Aarden LA, De Groot ER, Schaap OL, Lansdorp PM. Production of hybridoma growth factor by monocytes. *Eur J Immunol* 1987; **17**: 1411–1416.
17. Schindler R, Mancilla J, Endres S, Ghorbani R, Clark SC, Dinarello CA. Correlations and interactions in the production of interleukin-6 (IL-6), IL-1 and tumor necrosis factor (TNF) in human blood mononuclear cells: IL-6 suppresses IL-1 and TNF. *Blood* 1990; **75**: 40–47.
18. Millul V, Lagente V, Gillardeaux O *et al.* Activation of guinea pig alveolar macrophages by endothelin-1. *J Cardiovasc Pharm* 1991; **17(Suppl 7)**: S233–S235.
19. Burchett SK, Weaver WM, Westall JA, Larsen A, Kronheim S, Wilson CB. Regulation of tumor necrosis factor/cachectin and IL-1 secretion in human mononuclear phagocytes. *J Immunol* 1988; **140**: 3473–3481.
20. Elias JA, Schreiber AD, Gustilo K *et al.* Differential interleukin-1 elaboration by unfractionated and density fractionated human alveolar macrophages and blood monocytes: relationship to cell maturity. *J Immunol* 1985; **135**: 3198–3204.
21. Dinarello CA, Cannon JG, Wolff SM *et al.* Tumor necrosis factor (cachectin) is an endogenous pyrogen and induces production of interleukin 1. *J Exp Med* 1986; **163**: 1433–1450.
22. Aderka D, Le J, Vilcek J. IL-6 inhibits lipopolysaccharide-induced tumor necrosis factor production in cultured human monocytes, U937 cells, and in mice. *J Immunol* 1989; **143**: 3517–3523.
23. Johnson J, Meyrick B, Jesmok G, Brigham KL. Human recombinant tumor necrosis factor alpha infusion mimics endotoxemia in awake sheep. *J Appl Physiol* 1989; **66(3)**: 1448–1454.
24. Lembeck TF, Decrinis M, Pertl C, Amann R, Donnerer J. Effects of endothelin on the cardiovascular system and on smooth muscle preparations in different species. *Naunyn-Schmiedeberg's Arch Pharmacol* 1989; **340**: 744–751.
25. Sugiura M, Inagami T, Kon V. Endotoxin stimulates endothelin-release *in vivo* and *in vitro* as determined by radioimmunoassay. *Biochem Biophys Res Commun* 1989; **161**: 1220–1227.
26. Yoshizumi M, Kurihara H, Sugiyama T *et al.* Hemodynamic shear stress stimulates endothelin production by cultured endothelial cells. *Biochem Biophys Res Commun* 1989; **161**: 859–864.
27. Hieda HS, Gomez-Sanchez CE. Hypoxia increases endothelin release in endothelial cells in culture, but epinephrine, norepinephrine, serotonin, histamine and angiotensin II do not. *Life Sci* 1990; **47**: 247-251.

ACKNOWLEDGEMENTS. This work was carried out with grant support from Nordic Insulin Fond. The authors thank O. H. Olsen at the Blood Bank, University Hospital of Tromsø, for technical assistance with buffy coats, and W. Johnsen and Kristin Kjær for technical assistance with cytokine analysis.

**Received 9 July 1993; accepted in revised form 9 September 1993**
